# Cytogenetic analysis on geographically distant parthenogenetic populations of *Tityus trivittatus* Kraepelin, 1898 (Scorpiones, Buthidae): karyotype, constitutive heterochromatin and rDNA localization

**DOI:** 10.3897/CompCytogen.v8i2.6461

**Published:** 2014-03-12

**Authors:** Renzo Sebastián Adilardi, Andrés Alejandro Ojanguren Affilastro, Dardo Andrea Martí, Liliana María Mola

**Affiliations:** 1 Laboratorio de Citogenética y Evolución - Departamento de Ecología, Genética y Evolución, IEGEBA (CONICET-UBA), Facultad de Ciencias Exactas y Naturales, Universidad de Buenos Aires - Intendente Güiraldes 2160 - C1428EGA CABA, Argentina; 2 División de Aracnología - Museo Argentino de Ciencias Naturales “Bernardino Rivadavia” - CONICET - Ángel Gallardo 470 - C1405DJR CABA, Argentina; 3 Laboratorio de Genética Evolutiva - IBS (CONICET-UNaM) - Félix de Azara 1552 - CP3300 Posadas - Misiones, Argentina

**Keywords:** Scorpion, holokinetic chromosomes, parthenogenesis, karyotype, FISH, NOR

## Abstract

*Tityus trivittatus* Kraepelin, 1898 is the most medically important scorpion species of Argentina, and parthenogenetic populations are present in the major cities of this country. We performed a detailed cytogenetic analysis of specimens of three synanthropic parthenogenetic populations, all distant about 900 km from each other, using Ag-NOR, C-banding, DAPI/CMA_3_ staining and FISH with autologous 28S rDNA probes. The karyotype of females and embryos from the three populations showed 2n=6, with two large and four middle-sized holokinetic chromosomes. Constitutive heterochromatin was found in terminal and interstitial location and its pattern allowed the identification of three chromosome pairs. NORs were found on the terminal heterochromatic region of one pair of middle-sized chromosomes. The use of fluorochromes to characterize heterochromatin showed the absence of GC-rich heterochromatin and a low and variable number of AT-rich heterochromatic regions. We propose that a possible explanation for the lack of karyotypic variation between these geographically distant populations could be a recent colonization of urban areas by human means of synanthropic specimens from a single lineage of northeastern Argentina.

## Introduction

*Tityus* C. L. Koch, 1836 (Buthidae) is the most diversified genus of the order Scorpiones, with about 200 described species. It occurs from Central America to southern South America, in tropical and temperate areas. Several species of this genus are medically important, and most of the dangerous scorpion species in South America belong to the genus *Tityus* (Salomón and de Roodt 2001, de Roodt et al. 2003). This genus presents holokinetic chromosomes, as well as other genera of the family Buthidae, and a great intra- and interspecific variation of chromosome number, ranging from 2n=5 to 2n=27 (Schneider et al. 2009).

*Tityus trivittatus* Kraepelin, 1898 is the most medically important scorpion species of Argentina and it is responsible for several casualties (Maury 1997, de Roodt et al. 2010). It occurs in southern Brazil, Paraguay, and northern and central Argentina. It reaches Buenos Aires and La Plata cities, being the southernmost species of the genus. *Tityus trivittatus* became a synanthropic species in many areas, being present in most of the major cities of Argentina and Paraguay. *Tityus trivittatus*, as many species of the genus, is facultatively parthenogenetic; sexual populations were reported in Paraguay, southern Brazil and northern Argentina, however, Argentinean populations to the south of latitude 28°S are formed exclusively by parthenogenetic females (Maury 1997, Ojanguren Affilastro 2005).

Parthenogenesis in *Tityus* is quite common and besides *Tityus trivittatus* it has been mentioned to occur in several species, i.e. *Tityus columbianus* (Thorell, 1876), *Tityus confluens* Borelli, 1899, *Tityus metuendus* Pocock, 1897, *Tityus serrulatus* Lutz & Mello, 1922, *Tityus stigmurus* (Thorell, 1876) and *Tityus uruguayensis* Borelli, 1901 (Matthiesen 1962, Zolessi 1985, Toscano-Gadea 2005, Lourenço 2008, Ross 2010, Seiter 2012). Parthenogenesis was confirmed in *Tityus trivittatus* based on the observation of virgin females which could produce offspring after lifetime isolation in captivity (Toscano-Gadea 2005). Thelytokous parthenogenesis seems to be the principal mode of asexual reproduction in scorpions, except for the claim of arrhenotokous parthenogenesis in *Tityus metuendus*, which was severely disputed (Lourenço and Cuellar 1999, Francke 2008). Among these species, cytogenetic studies have been performed in parthenogenetic populations of *Tityus serrulatus* (2n=12) and *Tityus stigmurus* (2n=16), and in sexual populations of *Tityus confluens* (2n=13 in males), *Tityus metuendus* (2n=15 in males and females and 2n=16 in males) and *Tityus trivittatus* (2n=14 male), all from Brazil (Piza 1948, 1950, 1952, Schneider and Cella 2010, Mattos et al. 2013). However, we consider that the identity of the specimens of *Tityus trivittatus* and *Tityus confluens* from central Brazil analyzed in those studies is doubtful and should be confirmed with a deep taxonomic study of the group, since they have all been collected in areas placed far from the confirmed distribution of these species (Maury 1970, 1974, 1997, Murua et al. 2002, Fernández Campón and Lagos Silnik 2009). Records of *Tityus confluens* from central Brazil mentioned in Bertani et al. (2005) could probably belong to other closely related species.

In this contribution, we have cytogenetically studied specimens from three synanthropic parthenogenetic Argentinean populations of *Tityus trivittatus*, from Buenos Aires, Posadas, and Catamarca cities, all distant about 900 km from each other. The karyotype, constitutive heterochromatin distribution and composition, and ribosomal DNA localization were characterized.

## Materials and methods

We have studied females and embryos of *Tityus trivittatus* collected from urban populations at the cities of Buenos Aires (34°35.66'S, 58°24.68'W) and Posadas (Misiones province) (27°24.99'S, 55°55.96'W), both in Argentina. Seven females and eleven embryos (of four of these females), were collected by the authors in old subterranean tunnels below the children's Hospital “Dr Ricardo Gutiérrez”, placed in a highly urbanized area of Buenos Aires city. Nine females and seven embryos (of one of these females) were collected by the authors in a backyard of a house in the periphery of Posadas city, Misiones province. Also, two adult females (one of them with six embryos) were provided by the Department of Zoonoses of Catamarca province, Argentina. The exact locality of the specimens of Catamarca is unknown, but these specimens are most likely to have been collected in the city of San Fernando del Valle de Catamarca (28°28.14'S, 65°46.77'W), the biggest city of the province, where the Department of Zoonoses is placed.

All the specimens were carried alive to laboratory and killed by cooling down to -20°C. Their ovaries and embryos were dissected in saline solution (0.154 M NaCl), incubated in hypotonic solution (1:1 saline solution:distilled water) for 30 min, then fixed for 30 min in a freshly prepared Carnoy fixative (ethanol:chloroform:acetic acid, 6:3:1) and stored in fresh fixative. Pieces of ovaries or embryos were placed on slides and dissociated in a drop of 60% acetic acid with tungsten needles. Preparations with a drop of suspension were placed on a heating histological plate at 40–45°C; suspension was spread on the slides using a tungsten needle.

Conventional staining was made with 5% Giemsa solution in distilled water for 12–15 minutes. The C-banding was performed according to the protocol described by Sumner (1972) and stained with Giemsa or DAPI (4’-6-diamidino-2-phenylindole). The study of the nucleolar organizer regions (NORs) was made by silver-staining technique according to Howell and Black (1980). Fluorescent staining with DAPI and CMA_3_ (chromomycin A_3_) was carried out according to Rebagliati et al. (2003).

Ribosomal genes were detected by Fluorescence in situ hybridization (FISH) technique with 28S rDNA probe. Total genomic DNA of *Tityus trivittatus* was extracted using a DNeasy Tissue Kit (QIAGEN, Hilden, Germany). Unlabelled 28S rDNA probes were generated by PCR using primers 28Sa (5´-GACCCGTCTTGAAACACGGA-3´) and 28Sb (5´-TCGGAAGGAACCAGCTACTA-3´) (Whiting et al. 1997). The sequence of the 331bp fragment of the 28S rDNA gene was deposited in the NCBI database under the accession number KF723293. The probes were labelled by random primed labeling with DIG-11-dUTP using a DIG-High Prime labeling kit. FISH was performed as described by González et al. (2004) with slight modifications, and the probes were detected with Anti-digoxigenin-fluorescein Fab fragments (Roche Applied Science, Mannheim, Germany). The preparations were counterstained with DAPI and mounted in Vectashield (Vector, Burlingame, CA, USA).

To determine the karyotype, chromosome measurements of well-spread prometaphase cells from specimens of each population were made using Micro-Measure software, version 3.3 (Reeves and Tear 2000). The relative length of each chromosome was calculated as a percentage of total complement length (%TCL). This analysis was based on one female from Buenos Aires (10 cells), four embryos of one female from Posadas (10 cells), and one female (7 cells) and two embryos (10 cells) of another female from Catamarca. These data allowed us to prepare an idiogram.

## Results

**Figure 1.**
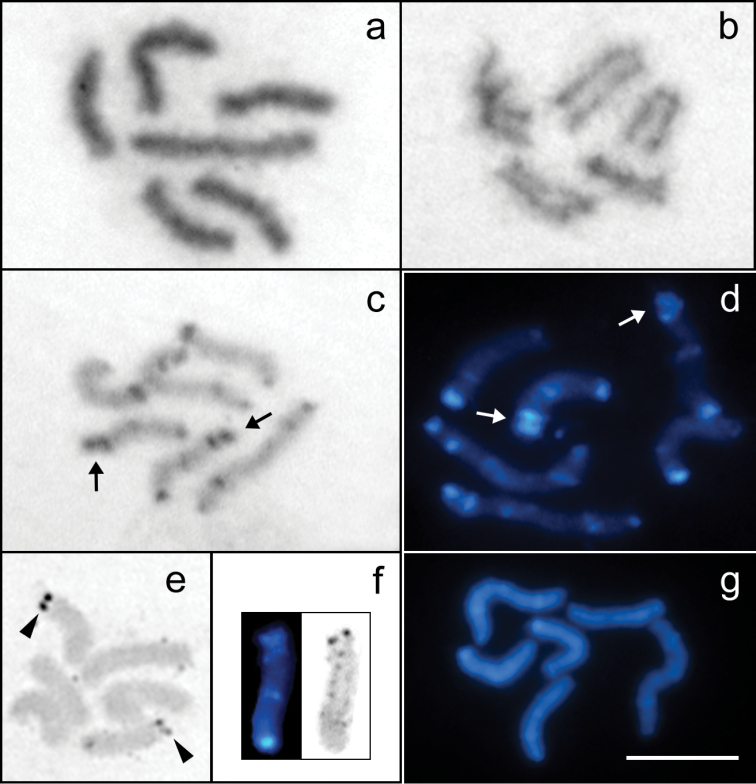
Mitotic cells of *Tityus trivittatus* (2n=6). **a** Giemsa-stained prometaphase **b** Early anaphase **c** C-banded prometaphase stained with Giemsa (Buenos Aires city) **d** C-banded prometaphase stained with DAPI (Buenos Aires city) **e** Silver-stained metaphase **f** Sequential C-banding and silver staining on chromosome 2 **g** DAPI-banded prometaphase after DAPI/CMA_3_ staining. The arrows point to the double-banded terminal region of pair 2. Arrowheads point to the NORs. Scale bar= 10 µm.

**Figure 2.**
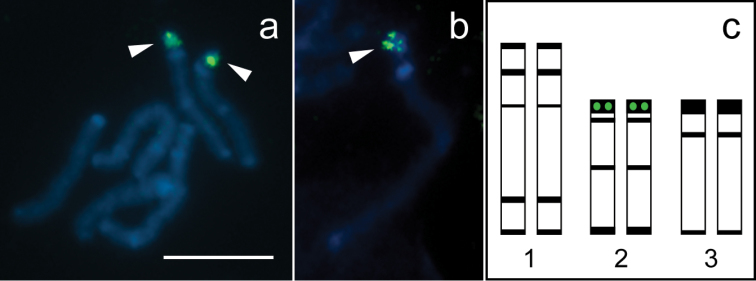
Fluorescence *in situ* hybridization with 28S rDNA probe and idiogram of the karyotype of *Tityus trivittatus*. **a** Mitotic prometaphase with hybridization signals **b** Chromosome 2 at late prophase with hybridization signal; **c.** Idiogram showing distribution of constitutive heterochromatin (black bands) and 28S rDNA clusters (green circles). Chromosomes are counterstained with DAPI (blue). Arrowheads point to hybridization signals (green). Scale bar= 10 µm.

The study of females and embryos of *Tityus trivittatus* from the parthenogenetic populations of Buenos Aires, Posadas, and Catamarca showed the same chromosome number of 2n=6, with two large and four middle-sized holokinetic chromosomes (Fig. 1a). Each large-sized chromosome presented an average value of 20.72% of the TCL, and the average value of the similar sized medium chromosomes was 14.64% of the TCL (Table 1) (Fig. 2c). The very few cells observed at early anaphase showed parallel arrangement of the sister chromatids, which is characteristic of holokinetic chromosomes (Fig. 1b).

**Table 1.** Chromosome measurements of the studied populations of *Tityus trivittatus*. Relative lengths expressed as percentage of total chromosome length (%TCL). Mean values and their standard deviations (SD) are given.

**Chromosome number**	**Buenos Aires**	**Catamarca**	**Posadas**
	**%TCL ± SD**	**%TCL ± SD**	**%TCL ± SD**
1	21.53 ± 0.99	21.54 ± 0.60	20.89 ± 0.79
2	19.96 ± 0.34	20.45 ± 0.65	19.92 ± 0.93
3	15.37 ± 0.57	15.44 ± 0.51	15.87 ± 0.55
4	14.93 ± 0.34	14.82 ± 0.45	15.07 ± 0.49
5	14.35 ± 0.33	14.28 ± 0.48	14.41 ± 0.63
6	13.86 ± 0.40	13.47 ± 0.55	13.84 ± 0.48

The study of specimens from the three localities revealed a complex pattern of C-bands with terminal, subterminal and interstitial localization, which made it possible to identify three chromosome pairs. This pattern was observed both with Giemsa and DAPI staining, although DAPI allowed a better resolution of smaller C-bands. The two large-sized chromosomes (pair 1) presented terminal and subterminal C-bands at each terminal region and one submedial band. The heterochromatic bands at one of the terminal regions are closer and the submedial band is located near of these bands. A pair of middle-sized chromosomes (pair 2) carried a C-band in one terminal region, a medial C-band and a conspicuous terminal and a subterminal C-band at the other terminal region. The other middle-sized chromosomes (pair 3) carried C-bands at each terminal region and a subterminal C-band (Figs 1c, d, 2c). In the specimens from Buenos Aires one of the terminal bands of pair 3 is more conspicuous and the subterminal band is closer to it.

Silver staining visualized active NORs at a terminal region of two middle-sized chromosomes (Fig. 1e). Cells with sequential C-banding and silver staining showed that NORs are located at the double-banded terminal region of pair 2 (Fig. 1f).

DAPI/CMA_3_ sequential staining revealed no bright CMA_3_ bands. Most cells showed chromosomes homogeneously stained with DAPI. Other cells showed some bright DAPI bands that were coincident with C-bands (Fig. 1g). The number of bright DAPI bands was less than the number of C-bands, and the smaller C-bands were not detected. This technique did not provide reliable results, since the number of DAPI bands was variable between cells with the same degree of chromosome condensation.

DAPI counterstaining in FISH technique revealed a similar pattern of bright bands as C-banding, which allowed for identification of each chromosome pair. Hybridization signals with the autologous 28S rDNA probes were located at the double-banded terminal region of pair 2 (Fig. 2a, c). Late mitotic prophase chromosomes revealed that the rDNA cluster is embedded in the conspicuous terminal C-band of pair 2 (Fig. 2b).

## Discussion

The chromosome number found in the specimens of the Argentinean populations of *Tityus trivittatus* herein studied is one of the lowest in Buthidae, and it is also present in *Tityus martinpaechi* Lourenço, 2001 and some individuals of *Tityus bahiensis* (Perty, 1834) (Piza 1939, Schneider et al. 2009, Mattos et al. 2013).

In other species of *Tityus*, Mattos et al. (2013) described two different patterns of heterochromatin distribution: species with small blocks of constitutive heterochromatin in the terminal regions of some chromosomes and species with more conspicuous blocks of constitutive heterochromatin in the terminal regions of all chromosomes and in the interstitial regions of some or all chromosomes. The specimens of *Tityus trivittatus* herein studied share the latter pattern of constitutive heterochromatin distribution.

The use of DAPI and CMA_3_ fluorochromes to characterize heterochromatin of *Tityus trivittatus* showed the absence of GC-rich heterochromatin and a low and variable number of AT-rich heterochromatic regions, which were coincident with some of the bands revealed by C-banding. In other Buthidae species the number of heterochromatic regions revealed by DAPI/CMA_3_ staining was also lower than that visualized by C-banding and these regions were almost exclusively AT-rich (only *Tityus martinpaechi* and *Rhopalurus agamemnon* (C. L. Koch, 1839) show GC-rich terminal regions in one chromosome pair) (Schneider and Cella 2010, Mattos et al. 2013). This difference could be related to the protocol of each technique: C-banding method implies a differential extraction of DNA that leads to a greater contrast between euchromatin and heterochromatin, whereas during direct DAPI/CMA_3_ staining there is no DNA extraction and the number of heterochromatic regions observed could be less (Sumner 2003, Barros-e-Silva and Guerra 2010). The low number of heterochromatic regions revealed with the latter technique could be also related to the holokinetic nature of the chromosomes, since it has been suggested that this type of chromosomes could be more rigid (Mandrioli and Manicardi 2012). A structural difference of the chromatin condensation of buthid mitotic chromosomes could hinder the specific fluorochrome binding to DNA.

The number and terminal location of NORs, as well as their association with constitutive heterochromatin found in the specimens of *Tityus trivittatus*, are all common features reported in other species of *Tityus* (Schneider et al. 2009, Schneider and Cella 2010, Mattos et al. 2013). Moreover, the terminal location of NORs is found in many other species of invertebrates and plants with holokinetic chromosomes (e.g.:Hemiptera, Lepidoptera, Odonata, Nematoda, Juncaceae, Cyperaceae and *Cuscuta* Linnaeus) (Albertson 1984, Rebagliati et al. 2003, Guerra and García 2004, Mola and Papeschi 2006, Criniti et al. 2009, Nguyen et al. 2010, Heckmann et al. 2011, Sousa et al. 2011, Poggio et al. 2011, Maryańska-Nadachowska et al. 2013), and this location of NORs could be a functional requirement to ensure chromosome stability in this type of chromosomes (Heckmann et al. 2011).

*Tityus trivittatus* is an invasive synanthropic species that easily colonizes urban areas due to its great adaptability, ubiquity and parthenogenetic reproduction. This species was probably introduced into Buenos Aires city during the first half of the twentieth century by anthropogenic means (Maury 1970). Parthenogenetic reproduction may allow the establishment of different karyotypes in isolated synanthropic populations. Nevertheless, all specimens of the three populations herein analyzed show the same karyotype in spite of the fact that the populations are about 900 km apart. The lack of variation between the studied populations could be due to a recent colonization of all these urban areas by specimens from a wild sexual (or even parthenogenetic) population with three pairs of homologous chromosomes. Another possible explanation is that all specimens from these cities belong to a single lineage that originally colonized cities from north-eastern Argentina, where its presence has been recorded long time ago (Werner 1902, Mello-Leitão 1934), and once it became synanthropic, specimens from these populations were easily transported from one city to another by human means. The last hypothesis seems more plausible, and is supported by the recent and fast colonization of all the cities of western Argentina (Murua et al. 2002, Fernández Campón and Lagos Silnik 2009), in areas that are far from the “Wet Chaco”, the original habitat of *Tityus trivittatus* (Ojanguren-Affilastro 2005). In two distant parthenogenetic populations of *Tityus serrulatus* with conserved karyotype, a particular combination of genes was proposed to have been selectively advantageous (Schneider and Cella 2010). This fact could also be related to the establishment of a particular karyotype in *Tityus trivittatus*.

Taking into account the high incidence of intra- and interpopulation chromosome rearrangements reported in other species of *Tityus*, further cytogenetic studies of unequivocally identified sexual and parthenogenetic populations of *Tityus trivittatus* are needed to reveal potential chromosome variation within this species.
